# Shedding light on Brazil’s contribution to photobiomodulation research in oral medicine: a bibliometric study

**DOI:** 10.1590/1807-3107bor-2025.vol39.052

**Published:** 2025-05-12

**Authors:** Lauren Frenzel SCHUCH, Laura Borges KIRSCHNICK, Vivian Petersen WAGNER, Vanessa Rodrigues VELHO, Gabriela Sauer LLANTADA, Marco Antonio Trevizani MARTINS, Márcia Martins MARQUES, Antonio Luiz Barbosa PINHEIRO, Jean Nunes dos SANTOS, Alan Roger SANTOS-SILVA, Cesar Augusto MIGLIORATI, Manoela Domingues MARTINS

**Affiliations:** (a) Universidad de La Republica - UDELAR, School of Dentistry, Department of Diagnosis in Pathology and Oral Medicine, Montevideo, Uruguay.; (b) Universidade Estadual de Campinas – Unicamp, Piracicaba Dental School, Department of Oral Diagnosis, Piracicaba, SP, Brazil.; (c) Universidade de São Paulo – USP, School of Dentistry, Department of Dentistry, São Paulo, SP, Brazil.; (d) Universidade Federal do Rio Grande do Sul – UFRGS, School of Dentistry, Department of Oral Pathology, Porto Alegre, RS, Brazil.; (e) Sigmund Freud University, Aachen Dental Laser Centre – AALZ, Austria Campus Prater, Vienna, Austria.; (f) Universidade Federal da Bahia – UFBa, School of Dentistry, Center of Biophotonics, Salvador, BA, Brazil.; (g) Universidade Federal da Bahia – UFBa, School of Dentistry, Laboratory of Oral and Maxillofacial Pathology, Salvador, BA, Brazil.; (h) University of Florida, College of Dentistry, Department of Oral & Maxillofacial Diagnostic Sciences, Gainesville, FL, USA.

**Keywords:** Bibliometrics, Low-Level Light Therapy, Stomatology

## Abstract

The aim of this study was to verify the role of Brazilian researchers in publications related to photobiomodulation (PBM) in the field of oral medicine. We examined ten years from 2012 to 2022 across 16 journals. Our analysis included scientific publications with a Brazilian author either in the first or last position and publications from international collaborations. The search yielded 43,525 publications. After examining titles and abstracts, 269 studies were categorized as having a specific emphasis on PBM in oral medicine, of which 147 (54.6%) were undertaken by research groups based in Brazil. The citations ranged from 1 to 149 (an average of 31 per manuscript). The male-to-female ratio of first and last author was 1:2 and 1:1.4, respectively. Brazilian involvement in the field of PBM was significant, with a marked focus on basic research, clinical applications, and technological advances. Our results also underscore the remarkable participation of female researchers in pivotal roles. Brazilian publications positively impacted healthcare worldwide using PBM in oral medicine, as evidenced by the substantial number of articles published and the citations of these articles received.

## Introduction

Photobiomodulation (PBM), also known as low-level laser therapy (LLLT) or laser phototherapy, is a therapeutic approach that involves the application of low-intensity light typically provided by lasers or light-emitting diodes (LEDs) to biological tissues to stimulate tissue growth, enhance wound healing, and manage pain. This non-invasive and non-thermal therapy is based on the principle that specific wavelengths of light can interact with cellular structures and molecules, leading to a series of photochemical and photophysical reactions within cells and tissues. In 1962, Endre Mester conducted groundbreaking experiments that laid the foundation for the emergence of PBM as a therapeutic modality.^1^ His work involved using low-intensity red laser light, and he initially intended to investigate the potential carcinogenic effects of laser radiation on skin tissues. Surprisingly, his experiments revealed the opposite: low-intensity laser irradiation stimulated skin healing and hair growth in mice, contrary to the expected detrimental effects. This serendipitous discovery marked the birth of PBM, also known as “laser biostimulation”, a novel approach in medical science. However, back in 1903 Niels Ryberg Finsen had already applied red and blue light to treat human diseases, especially lupus, showing that concentrated sunlight was able to kill bacteria and stimulate nearby tissues.^2^ For his findings, Finsen was awarded the Nobel Prize in Medicine and Physiology.^3,4^

PBM gained recognition in various medical specialties, including dermatology, physiotherapy, sports medicine, and dentistry. It treats multiple conditions, such as chronic pain, inflammation, and skin disorders. In oral medicine, compelling evidence indicates the use of PBM, including for facial pain and neuromuscular disorders (e.g., orofacial pain and temporomandibular disorders), dermatologic diseases (e.g., lichen planus and pemphigus vulgaris), burning mouth syndrome, xerostomia/hyposalivation, chemo- and/or radiation-induced oral mucositis (OM), recurrent herpes simplex, recurrent herpes labialis, and recurrent aphthous ulcerations/stomatitis.^5-14^ In a field where many clinical decisions are still based on expert consensus, PBM is one of the most evidence-based approaches for some oral conditions.^11,15,16^ As a result, PBM has been added to international guidelines, such as the MASCC/ISOO mucositis guidelines.^11,17,18^

Despite the challenges posed by Brazil’s status as a middle-income nation undergoing notable cuts in science funding, it is noteworthy that a significant volume of publications on PBM therapy originates from this country.^19^ The study by Gonçalves et al. investigated the prevalence of co-authorship of Brazilian researchers in distinguished dental journals, and found that Brazil is the second most productive contributor globally.^20^ Farias and collaborators in their investigation of articles on oral pathology, oral surgery, and oral medicine underscored the influence of Brazilian researchers in the international research arena.^19^ Notably, a recent article commemorating 50 years of stomatology in Brazil highlighted the pivotal role played by Brazilian research in advancing global understanding, education, and innovations in oral medicine.^21^ In the realm of PBM, the extensive Brazilian landscape emphasized by Dr Hamblin underscores the importance of elucidating its contributions to the scientific community.^22^ Within this context, our primary aim was to assess the prevalence of research articles authored by Brazilians in relevant academic journals addressing the application of PBM in oral medicine.

## Methods

### Search process

This bibliometric study was based on records from 16 peer-reviewed journals in oral medicine and PBM. The journals were selected based on the following criteria: a) Journals indexed in Clarivate Analytics’ Journal Citation Reports (JCR); b) Journals with a focus on or significant sections dedicated to oral medicine and PBM.

The issues of the selected journals were manually retrieved for the period between January 2012 and December 2022. One author reviewed the tables of contents of each issue to identify articles concerning PBM in oral medicine. The review process occurred two steps. First, the titles and abstracts were thoroughly reviewed to ensure that they fell within the field of PBM in oral medicine. Second, the authors’ names and affiliations were scrutinized to identify studies authored by individuals of Brazilian origin.

### General data collection,

The following data was collected: a) name of the journal; b) total number of published articles; c) total number of published PBM articles; d) Brazilian scientific PBM articles. The respective Brazilian regions (i.e., North, Northeast, Central-West, Southeast, South) and the gender of the first and last author were obtained based on the authors first name.

### Type of study

The study design was retrieved from the article’s title and/or abstract.

### Funding information and international collaboration

Brazilian publications with international partners were rated Yes or No based on the presence of a co-author with non-Brazilian affiliation. The funding information was also retrieved.

### Metric data collection

For each evaluated journal, metrics were collected from the Clarivate Analytics Journal Citation Reports (JCR) platform. The analysis of this study was centered on citation data reported in the 2022 JCR. The following information was acquired: a) total citations; b) 5-year impact factor - IF; c) rank by journal impact factor - JIF; d) rank by journal citation information – JCI. The number of citations of Brazilian articles was collected through Google Scholar.

### Statistical analysis

Statistical Package for the Social Sciences (SPSS) software, version 20.0 (IBM Corporation, Armonk, USA), and GraphPad software, version 10.0 (GraphPad Software, San Diego, CA), were used for analysis. Non-parametric tests, including the Mann-Whitney and Kruskal-Wallis tests, were used to analyze quantitative variables (i.e. number of citations, year of publication) in relation to categorical variables (i.e., journal type, specific journals, study type, and presence of international collaboration). The Spearman correlation test was applied to assess correlations between quantitative variables. A p-value < 0.05 was considered statistically significant for all tests.

## Results

### General data

**Figure 1.**
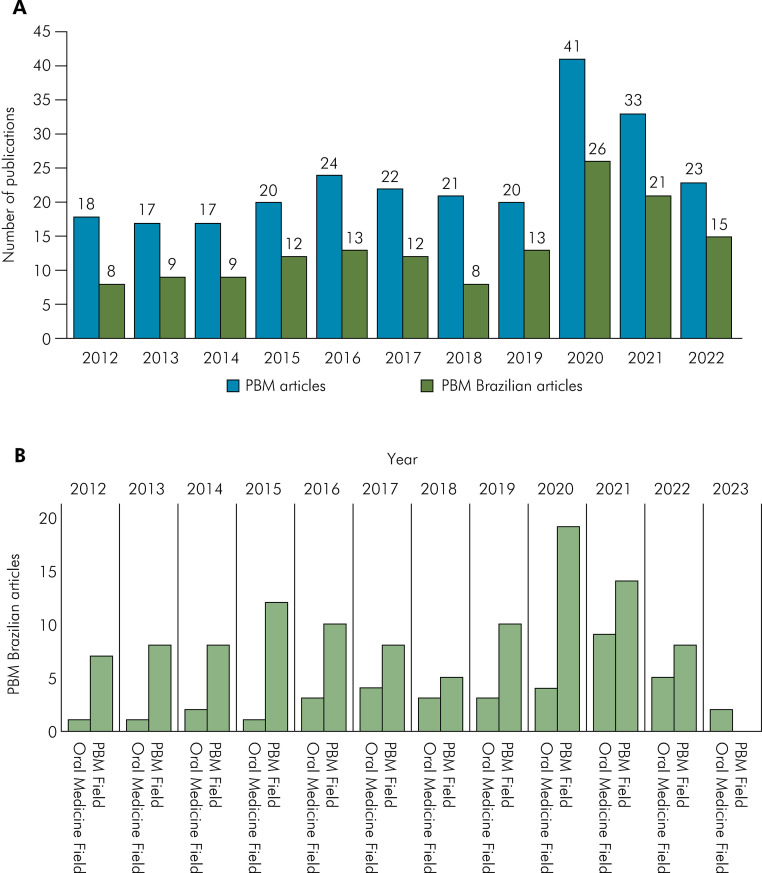
A) Distribution of PBM articles (total and Brazilian) in 10 years. B) Distribution of Brazilian articles in journals in the fields of PBM and Oral Medicine.

The search in the 16 journals yielded 43,525 publications (Table). Among them, 269 (0.6%) were articles on PBM of which 147 (54.6%) were from Brazilian authors. Figure 1 depicts these publications by year, showing the number of all articles published and the ones with the participation of Brazilian authors (Figure 1A). Every year, Brazilian publications account for approximately 50% of the publications. In all the periods analyzed, the absolute number of publications was higher in PBM-specific journals compared to general oral medicine journals (Figure 1B).

**Table.** Brazilian scientific publications on photobiomodulation in oral medicine in a period of 10 years.

Scientific journal	Journal Citation Reports				Total number of articles published	Total number of PBM articles published	Brazilian scientific PBM articles	Brazilian region					Gender (first/last author)	
	Journal’ Total Citation	5-year impact factor	Rank by JIF	Rank by JCI				North	Northeast	Central-West	Southeast	South	Male	Female
Oral medicine field														
Journal of Oral Pathology & Medicine	6,384	3.5	30/91	25/156	1,431	6 (0.4%)	0 (0)	0	0	0	0	0	0 / 0	0 / 0
Medicina Oral, Patologia Oral y Cirurgia Bucal	4,019	2.6	61/91	65/156	1,238	8 (0.6%)	3 (37.5%)	0	3	0	0	0	0 / 3	3 / 0
Oral Diseases	8,346	3.6	19/91	30/156	1,837	10 (0.5%)	4 (40%)	0	0	0	3	1	1 / 0	03/abr
Oral Oncology	13,846	4.9	ago/91	29/156	3,311	7 (0.2%)	5 (71.4%)	0	1	0	4	0	04/abr	01/jan
Oral Surgery, Oral Medicine, Oral Pathology, Oral Radiology	15,765	2.5	40/91	46/156	2,933	6 (0.2%)	1 (16.7%)	0	0	0	1	0	01/jan	0 / 0
Brazilian Oral Research	3,364	3.0	49/91	58/156	1,305	6 (0.4%)	4 (66.7%)	0	0	0	3	1	1 / 0	03/abr
Archives of Oral Biology	10,232	2.8	37/91	25/156	2,578	7 (0.2%)	2 (28.6%)	0	0	0	2	0	01/jan	01/jan
Supportive Care in Cancer	21,042	3.5	51/105	53/166	6,19	38 (0.6%)	16 (42.1%)	0	4	1	10	1	03/out	13/jun
Clinical Oral Investigations	13,908	3.5	28/91	19/156	4,114	4 (0.1%)	3 (75%)	0	1	1	1	0	0 / 0	03/mar
Photobiomodulation field														
Photodiagnosis and Photodynamic Therapy	7,474	3.3	135/241	165/317	2,654	23 (0.8%)	17 (73.9%)	0	5	0	12	0	05/abr	dez/13
Lasers in Medical Sciences*	944	1.7		147/202	2,666	79 (2.9%)	47 (59.5%)	0	7	3	30	7	17 / 14	29 / 32
Photochemical & Photobiological Sciences	8,148	3.4	173/285	157/315	2,111	3 (0.1%)	3 (100%)	0	0	0	3	0	02/fev	01/jan
Journal of Photochemistry and Photobiology B: Biology	18,082	6.0	73/285	42/315	3,025	18 (0.6%)	12 (66.7%)	0	1	0	7	4	03/set	09/mar
Journal of Biophotonics	5,494	2.8	48/77	36/83	1,95	8 (0.4%)	6 (75%)	0	0	0	5	1	02/fev	04/abr
Photomedicine and Laser Surgery	3,542	2.9	83/211	106/271	1,024	35 (3.4%)	15 (42.8%)	0	2	1	11	1	07/jul	08/ago
Journal of Biomedical Optics	14,729	3.2	27/77	23/83	4,049	11 (0.2%)	9 (81.8%)	0	0	0	2	7	01/mar	08/jun
Total	-	-	-	-	43,525	269 (0.6%)	147 (54.6%)	0 (0%)	24 (16.3%)	6 (4.1%)	94 (64%)	23 (15.6%)	48 / 60	98 / 86

PBM: photobiomodulation; JIF: journal impact factor; JCI: journal citation indicator. *One study did not specify the author’s sex.

### Types of study design

**Figure 2.**
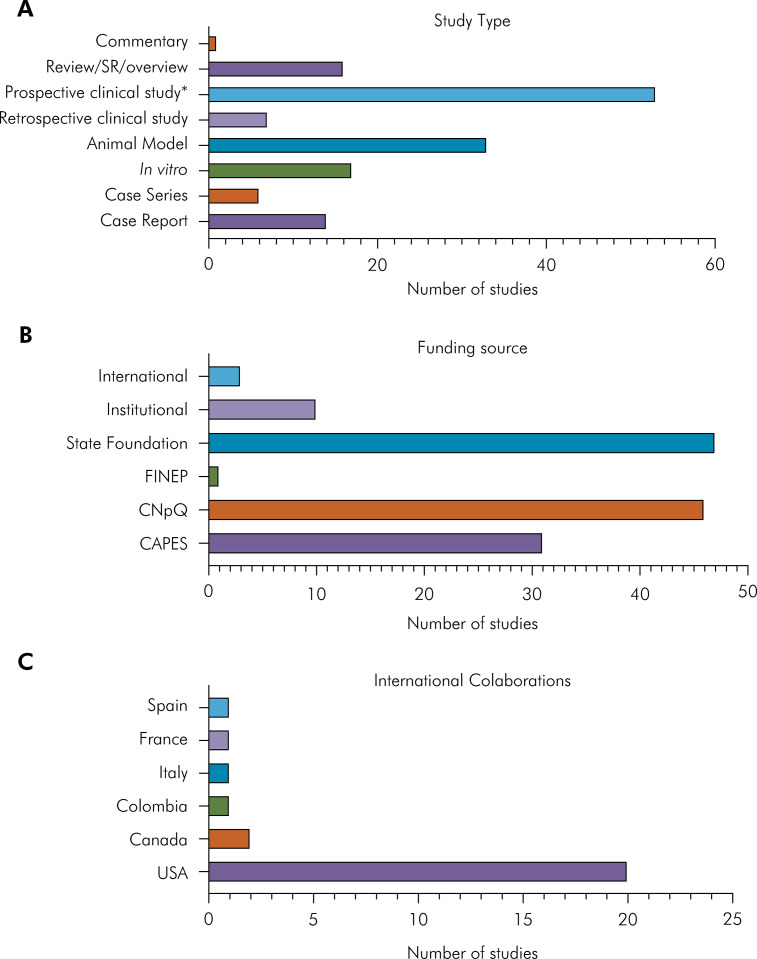
A) Number of Brazilian PBM publications according to study type. B) Disclosed funding sources of Brazilian PBM publications. C) Country of origin of international collaborators in Brazilian PBM publications.

The articles included case reports, case series, *in vitro* and animal model studies, clinical, retrospective, and prospective studies (clinical trial, case-control, and cross-sectional), reviews, and commentaries. The distribution of studies is disclosed in Figure 2A.

### Funding information and international collaboration

Funding information was disclosed in 83 studies (Figure 2B) and comprised mainly Brazilian national funding agencies, such as the Brazilian Ministry of Education Foundation (Capes) (n = 31), the Brazilian National Council for Scientific and Technological Development (CNPq) (n =4 6), and Finep (n = 1), which are under the Ministry of Science and Technology. State foundations were mentioned in 47 studies, and included the following states: São Paulo (n = 34), Minas Gerais (n = 4), Goiás (n = 4), Rio Grande do Sul (n = 2), Sergipe (n = 1), Maranhão (n = 1), and Rio de Janeiro (n = 1). Other sources disclosed were national institutional funding (n = 10) and international agencies or institutions (n = 3). Several studies disclosed more than one funding source.

International collaborations were observed in 24 publications with 6 countries. The cooperation with USA authors was the most prominent, followed by Canadian authors (Figure 2C).

### Publication trends, regional representation, and institutional affiliations

**Figure 3.**
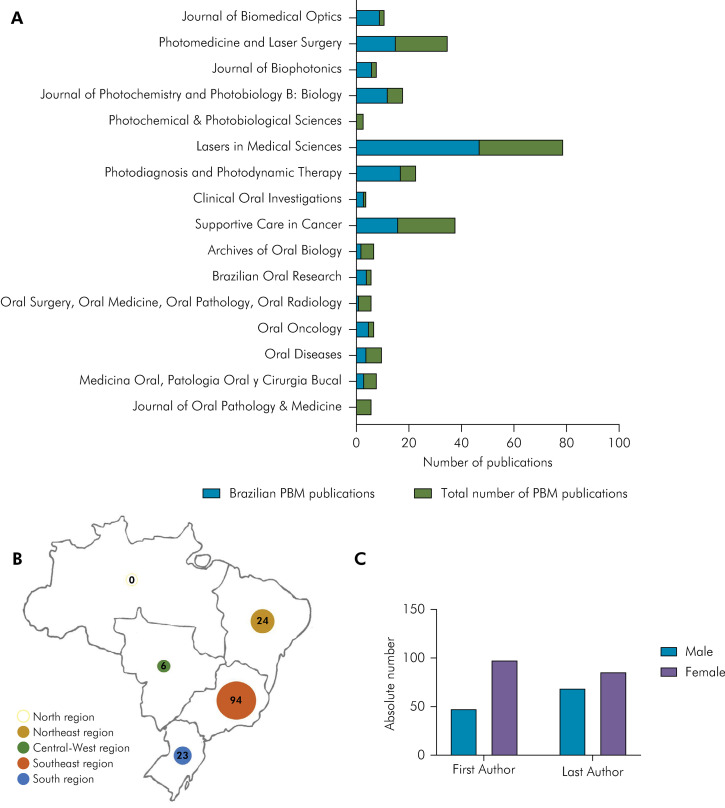
A) Total number of PBM publications (blue) and Brazilian representation (green) amongst each journal. B) Number of studies in each geographic region. C) Gender distribution of first and last authors.

Publications on PBM in oral medicine were found in 16 different journals. Most were oral medicine journals and 7 (43%) were journals specialized in laser. The journal “Lasers in Medical Sciences” published the most studies, followed by “Supportive Care in Cancer” and “Photomedicine and Laser Surgery” (Figure 3A).

Concerning Brazilian regions, authors of the Southeast region (64%) published the most papers (Figure 3B). In the authorship hierarchy, the male-to-female ratio for the first and last author was 1:2 and 1:1.4, respectively (Figure 3C).

An analysis of the affiliations of the first and last authors across various journals revealed significant representation of multiple Brazilian institutions. Notably, the Universidade de São Paulo (USP) appeared frequently in journals such as Oral Diseases, Oral Oncology, and Photodiagnosis and Photodynamic Therapy. Other prominent universities included Universidade Estadual de Campinas (UNICAMP), Universidade Federal do Rio Grande do Sul (UFRGS), and Universidade Federal da Bahia (UFBA), with notable contributions from Universidade Federal de Pernambuco (UFPE) and Universidade Federal da Paraíba (UFPB) in Medicina Oral, Patologia Oral y Cirurgia Bucal. Additionally, the Instituto Nacional do Cancer (INCA) was involved in Oral Oncology, and private institutions such as A.C. Camargo Cancer Center and Christus University Center contributed to research in photodynamic therapy.

### Metric characteristics

**Figure 4.**
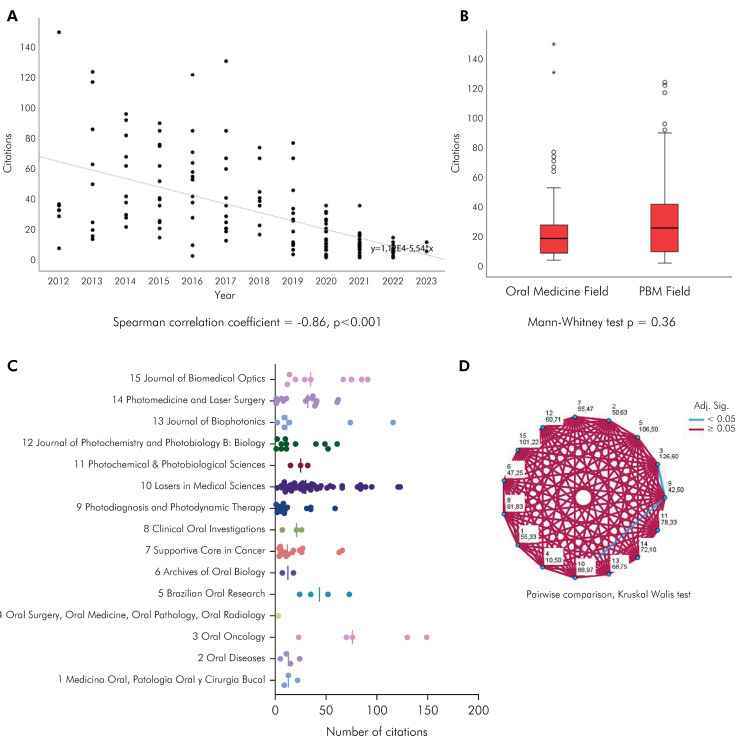
A) Scatter plot showing a significant inverse correlation between number of citations and year of publication. Older articles had the highest citation scores. B) Box plot of number of citations according to type of journal (Oral medicine vs PBM), showing a tendency for higher citation in PBM journals. C) Interleaved scatter of number of citations according to journal. D) Pairwise comparison in which red lines denote no significant difference and blue lines statistically significant differences. These were observed between Laser in Medical Science vs Photodiagnosis and Photodynamic Therapy and Oral Oncology vs Photodiagnosis and Photodynamic Therapy.

Google Scholar displayed the highest mean citation count of Brazilian publications with 31.84 citations (range: 1–149). Scopus had a mean of 20.09 citations (range: 0-95), while Web of Science showed a mean of 18.66 citations (range: 1-90). As expected, the articles with the most citations were published for extended periods, as evidenced by a scatter plot (Figure 4A). When only studies with more than five years since its publication were analyzed, the overall mean was 49.89 citation per article. There was a tendency for higher citation numbers for studies published in PBM journals, but this was not statistically significant (Figure 4B). The number of citations according to journal revealed a non-homogenous pattern (Figure 4C). Regarding Brazilian PBM publications, the pairwise comparison showed that the journal “Laser in Medical Sciences” (Impact Factor 1.7) and “Oral Oncology” (Impact Factor 4.9) showed significantly higher citation numbers compared to those of the “Photodiagnosis and Photodynamic Therapy” (Impact Factor 3.3) (Figure 4D).

**Figure 5.**
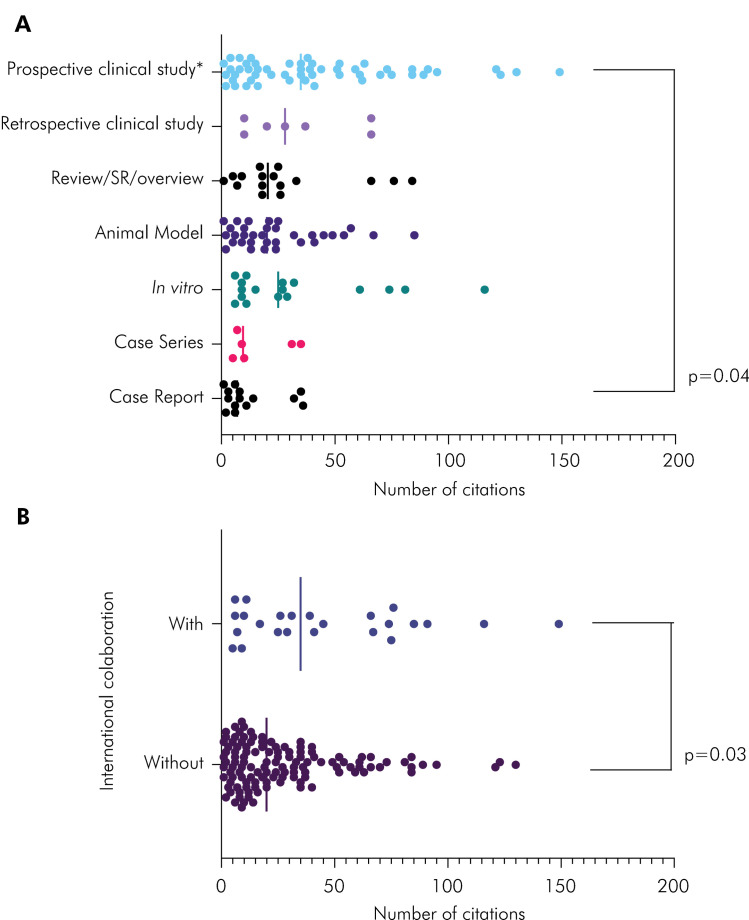
A) Interleaved scatter of number of citations according to study type. Prospective clinical studies had more citations than case reports (Dunn Multiple comparison test). Other pairwise comparisons were not significant. B) Interleaved scatter of number of citations according to international collaboration. Mann-Whitney test showed that studies which involved international partners had more citations.

The number of citations according to study type also varied (Figure 5A). Prospective clinical studies, which included clinical trials, case-control, and cross-sectional studies, had more citations than case reports (Dunn Multiple Comparison Test, p = 0.04). The other comparisons showed no statistically significant results despite the tendency of higher citation numbers for *in vitro* and retrospective clinical studies. Comparing the number of citations according to international collaborations, studies that involved international partners had higher citation scores than exclusively Brazilian studies (Figure 5B) (Mann-Whitney test, p = 0.03).

## Discussion

Our findings demonstrated a significant participation of Brazilians in the scientific production related to PBM in the oral medicine field, being responsible for more than 50% of the publications analyzed. Several factors explain this prominent role in disseminating PBM knowledge, including the quantity and quality of dental research, Brazil’s pioneering application of lasers in dentistry, governmental incentives, international collaborations, and equipment developed by Brazilian engineering institutions, among other aspects.

Bibliometric studies are valuable resources for assessing the output of researchers in a particular field, gauging the impact of funding initiatives, and assessing the contributions of various regions to the global research landscape.^23^ Quantitative data also provide transparency and accountability for allocated resources and can guide future investments.

Brazil has a dental workforce of around 402,907 practitioners.^24^ Brazilian dental research has made significant strides in the quantity and quality of research output and its impact on the global dental community.^19^ The prominent female leadership in Brazilian publications on PBM in oral medicine must be acknowledged. The presence of a greater number of female students in both undergraduate and postgraduate programs may contribute to their growing presence inr higher hierarchical positions within the system. Recently, an examination of the involvement of women in articles published in high-impact factor dental journals revealed that women’s representation was notably limited, indicating a substantial gender disparity in scientific output.^25^ This disparity extends globally, where women’s participation in scientific publications across various fields is lower than men’s.^26^ In 2021, Quintão et al. published an extensive discussion exploring gender disparities in Orthodontics.^27^ Also, the authors provided a general landscape to track women’s progress in managerial and leadership positions within academic and professional spheres. They posit that the integration of women into the educational sphere began significantly later than that of men. Therefore, looking at the progress and evolution of female participation over time, it is evident that they are increasingly and consistently occupying spaces within this domain. In a study of 22 high-standard postgraduate dentistry programs in Brazil (i.e., with Capes/2017 scores of 5–7), 63.64% were led by women and 36.36% by men.^27^It is important to note that in this study, gender was assessed based on the authors’ first names. While this approach posed no ambiguity in our dataset, which included only Brazilian names, it assumes conventional gender associations and may not account for non-binary or non-traditional gender identities. Although self-reported gender data would improve accuracy, such data was not available for our study, which may represent a source of bias.

The pioneering in PBM is mainly due to the work of eminent researchers who initiated this subject a few decades ago.^28-32^ Their universities and research institutions established dedicated research centers and laboratories to investigate the effects of PBM. Therefore, Brazilian researchers and engineers have developed advanced laser and LED devices for PBM therapy.^33^ These innovations include portable and user-friendly devices that can be used in clinical settings.

Greater ownership of scientific information is essential to stimulate an evidence-based clinical practice. In addition, governmental incentives for technological innovation are fundamental to technological development.^34,35^ Of the 147 Brazilian papers, funding information was disclosed by 83 studies, mostly from national funding agencies. Capes Foundation and CNPq were acknowledged as funding sources in 31 and 46 studies, respectively. Capes funding is mainly associated with student scholarships, and is the primary source of master’s and PhD funding in Brazil. This highlights the vital role of graduate students in developing scientific data. CNPq funding comprises both research grants and fellowships for Brazilian principal investigators. This agency awards researchers with outstanding scientific productivity. Our findings confirm that these national governmental investments are essential and are associated with high-quality scientific output.

Geographical analysis revealed that the Southeast region had the most significant scientific production, which was directly associated with funding resources. The state of São Paulo was highly represented in the allocation of funds through its Fapesp foundation. In addition, 72% of the studies with regional funding were from São Paulo, with Fapesp significantly contributing to science by providing research grants, scholarships, and funding for various research projects and initiatives. While this highlights the strengths and excellence of this foundation, it also demonstrates the inequity in the country.

Most publications were of clinical studies, indicating that Brazilian researchers have conducted numerous clinical trials to evaluate the efficacy of PBM in various medical and dental applications. Brazil has established regulatory guidelines and standards to ensure that PBM devices are safe and effective. The Brazilian Health Regulatory Agency (Anvisa) oversees the registration and approval of medical devices used in PBM therapy. Educational programs and workshops have also been developed to train healthcare professionals in the proper use of PBM devices.

The studies on PBM applied to oral medicine have been published in several journals, and only 43% of these studies were published in specialized laser journals. This finding shows that PBM therapy has been presented to general oral medicine practitioners and not only to professionals in the laser field. Generally, all studies with more than 5 years of publication had a substantial number of citations. The four most cited studies were published between 2012 and 2017, and all were clinical investigations within the domains of osteoradionecrosis,^36^ oral mucositis,^37^ and temporomandibular disorders.^38,39^ Paschoal et al.^40^ bibliometric analysis of the 100 most-cited papers focusing on laser applications in dentistry found that Brazil was the exclusive representative from Latin America, with this active participation credited to specialized centers such as the Special Laboratory of Laser in Dentistry (LELO, in Portuguese) at the University of São Paulo, collaborating with the Nuclear and Energy Research Institute. The robust partnerships between LELO and Japanese and German institutions underscored the excellence of Brazilian research, evident in its 7th and 8th positions among the top 100 most-cited papers. LELO researchers collaborated with international experts in the field of PBM, contributing to the global body of knowledge in this area. Our findings also demonstrated a high number of American collaborations in the analyzed articles. This has resulted in the exchange of research findings and best practices.

The quantitative insights of this investigation have the potential to influence future resource allocation strategies while providing valuable data for researchers, policy makers, and industry stakeholders. These data underscore Brazil’s prominent role and contribution in the arena of PBM in oral medicine.

## Conclusion

Brazilian research has played a significant role in the advance of the understanding of PBM in oral medicine. Furthermore, the presence of female researchers in pivotal roles was remarkable. To emphasize the critical nature of this matter beyond the number of publications, the discernible quality of these studies was evident through the meticulous analyses of citations and journal metrics. The achievements in this field are due to influential factors, including substantial funding support and active international collaborations.
